# Experimental Induction of Mammary Carcinogenesis Using 7,12-Dimethylbenz(a)anthracene and Xenograft in Rats

**DOI:** 10.7759/cureus.77648

**Published:** 2025-01-19

**Authors:** Adamantia Kontogeorgi, Ioannis Boutas, Nektarios I Koufopoulos, Dionysios T Dimas, Kyparissia Sitara, Antonios Makrigiannakis, Dimitrios Goutas, Sophia Kalantaridou

**Affiliations:** 1 Medical School, University of Crete, Heraklion, GRC; 2 Breast Unit, Rea Maternity Hospital, Athens, GRC; 3 Second Department of Pathology, School of Medicine, Attikon University Hospital, National and Kapodistrian University of Athens, Athens, GRC; 4 Breast Unit, Athens Medical Center, Psychiko Clinic, Athens, GRC; 5 Department of Internal Medicine, Elpis General Hospital of Athens, Athens, GRC; 6 Department of Obstetrics and Gynecology, University of Crete, Heraklion, GRC; 7 Department of Pathology, National and Kapodistrian University of Athens, Athens, GRC; 8 Third Department of Obstetrics and Gynecology, School of Medicine, National and Kapodistrian University of Athens, Athens, GRC

**Keywords:** animal model, biomodulators, experiment, mammary carcinogenesis, molecular mechanisms, ovariectomy, rats, xenograft

## Abstract

Background: Basic research using animal models is essential for investigating pathogenic molecular mechanisms and exploring new pharmaceutical treatments for tumors, including breast tumors.

Methods and results: Thirty-six female Wistar rats, four weeks old, were included in the study, but six were excluded early due to health complications, leaving 30 rats in the final cohort. Mammary gland tumors were induced using a single subcutaneous dose of 20 mg 7,12-dimethylbenz(a)anthracene (DMBA) on the right side and MCF7 cells (xenograft) on the left thoracic gland. The experiment spanned 28 weeks. Blood and white cell counts were monitored at the start, mid-point, and end of the study. Tumorigenesis was achieved via both DMBA and MCF7 xenograft. Ovariectomy was performed on all rats, and tumors became palpable after the fifth week following DMBA and MCF7 administration.

Conclusions: Oncogenesis using DMBA and MCF7 xenograft simultaneously had not been previously attempted. The results showed a 16.7% (6/36) animal mortality rate in the early stages of experimentation and a 100% induction of palpable tumors in the surviving animals.

## Introduction

Breast cancer is the most common neoplasm in women, accounting for one-third of newly diagnosed oncological cases annually [1]. In developed countries, breast cancer is the second most frequent cause of cancer-related mortality after lung cancer [2], while in developing countries that have been traditionally known for low incidence, there has been an increase in both incidence and mortality recently [3]. Epidemiological observations have shown that continuous research and development in the field of primary and secondary prevention, along with research in the field of treatment, are necessary. This is required to allow the scientific community to protect these women by giving them the possibility of early diagnosis and treatment for this neoplasia [4].

In recent years, the potential use of biomodulators to influence breast carcinogenesis has shown great promise. This includes agents such as aromatase inhibitors, cyclooxygenase inhibitors, selective estrogen receptor modulators (SERMs), and dietary factors, which offer exciting avenues for prevention and treatment strategies [5]. Furthermore, the study on the progesterone receptor has shown that it can be a promising research subject, while many new research protocols include the use of first-, second-, and third-generation progesterone receptor inhibitors [6-8]. However, before proceeding to clinical investigations, it is essential that all these strategies undergo thorough validation in animal models to ensure their safety and efficacy.

Basic research in animal models at this stage is decisive both for the investigation of pathogenic molecular mechanisms and for the exploration of new pharmaceutical options for the treatment of tumors [5]. The selected models should closely resemble human breast cancer in terms of pathology, biological behavior, tumor etiology, and response to therapeutic interventions [9]. However, it is important to acknowledge that no animal model can perfectly replicate all aspects of human breast cancer. Its unpredictable nature is because it involves living organisms, so each experimental setup is expected to have advantages and disadvantages. The high heterogeneity and complexity of breast tumors also contribute to the already perplexed nature of this type of experiment [10]. These considerations have led to the development of various animal models, each simulating specific aspects of human breast cancer. Consequently, it is essential to use a combination of models to gain a comprehensive understanding of breast cancer biology and to advance the development of effective prevention and therapeutic strategies [9].

For selecting the correct specimen of animal species, mice and rats remain the most common choice [11,12]. To achieve breast tumorigenesis, these animals are treated with physical, biological, and chemical carcinogens that are administered via injection, orally, or through whole-body treatments [12]. The most widely used method for inducing cancer in animal models is the administration of 7,12-dimethylbenz(a)anthracene (DMBA) [13]. The administration is usually intravenous, subcutaneous, or intragastric. DMBA rat breast cancers are mostly hormone-dependent [14]. Another approach is transplantation, which involves implanting spontaneous or induced breast cancer tissues or cells into animals. This method allows researchers to study tumor behavior and response to treatments in a controlled environment [15,16]. Transplantation models can be classified into allograft and xenograft models, depending on the source of the transplant. Xenograft models, which involve the transplantation of human cancer cells or tissues into immunodeficient mice, are particularly significant. They remain the primary tool for therapeutic drug discovery and evaluation, offering valuable insights into tumor behavior and treatment responses [17-19].

The purpose of this study is to investigate tumorigenesis in rats using a combination of DMBA and xenograft. This is the first research effort attempting to combine the two methods to grow tumors for further pharmacological research.

## Materials and methods

Ethics statement

All animal protocols used in this study adhered to the guiding principles of laboratory animal care as outlined in NIH Publication No. 85-93 (revised 1985) [20]. The Athens Prefecture officially approved the protocols under the Protocol for Experimental License. All experimental setups and studies were conducted at the Laboratory of Experimental Anatomy "Christeas" at the National and Kapodistrian University of Athens, Greece, under the supervision of qualified veterinary staff. The care and use of animals in this study complied with the approved animal welfare laws, guidelines, and policies of the European Union and Greece, as outlined in Law 2015/1992 (Official Gazette 30/A/27.2.1992), including the ratification of the European Convention for the Protection of Vertebrate Animals used for experimental or other scientific purposes.

Animals and conditions

Thirty-six female, four-week-old Wistar rats were acquired from the Laboratory of Experimental Anatomy "Christeas," National and Kapodistrian University of Athens, Greece. Throughout the acclimatization and experimental periods, the rats were housed in polycarbonate cages, with no more than three rats per cage. The housing environment was maintained under controlled conditions, including a temperature of 22.2 (±1.7) °C, humidity of 48.4 (±6.0%), and a 12-hour light/dark cycle. The rats were provided with rodent pellets and water ad libitum. Tumorigenesis was induced in all animals via DMBA and MCF7 cancer cells using the xenograft approach.

7,12-Dimethylbenz(a)anthracene

Mammary gland tumors on the right side of the rats were induced by administering a single subcutaneous dose of 20 mg of DMBA, diluted in 1 mL of soy oil. The rats, with an average weight of 195 g (range: 161-213 g), received the chemical carcinogen at 80 days of age.

Cell culture

The necessary cell culture materials, including Modified Eagle Medium (MEM), trypsin, 100× penicillin-streptomycin-fungizone (PSF), phosphate-buffered saline (PBS), and fetal calf serum (FCS), were procured from Whitehead Scientific (Cape Town, South Africa). Human breast adenocarcinoma cell lines (MCF7) were obtained from the American Type Culture Collection (ATCC, Manassas, VA, USA). These adherent cells were cultured in MEM cultures supplemented with 10% FCS and 1-2% PSF.

A vial of Basement Membrane Extract (BME) with a concentration of 3.158 mg/mL was stored at -80 °C. The vial was retrieved from storage and placed on ice to thaw over 24 hours prior to tumor induction. Monolayer-cultured cells were trypsinized at passage two and quantified to achieve a total cell density of 7.2 × 10⁶ cells/mL. The injection preparations consisted of equal volumes of MCF7 cells and BME. Using the xenograft method, a 22-gauge needle was used to carefully inject 1 mL of the preparation, containing either MCF7 cells alone or a mixture of MCF7 cells and BME, subcutaneously into the left thoracic gland of the rats.

Hematological analysis

On the day of DMBA administration and xenograft-based cell line induction, each rat was placed in a warming chamber for a few minutes before blood collection. Blood samples (1 mL) were drawn from the lateral tail vein using a 22-gauge needle and collected in heparinized vacutainers. To ensure accuracy during necropsy, a 12-hour fasting period was maintained prior to the procedure. On the 20th day of the experiment, the animals were anesthetized with an overdose of halothane, followed by immediate cardiac puncture for exsanguination. Blood samples were again collected in heparinized vacutainers, and white and red blood cell counts were analyzed using a Beckman Coulter DXC600/800 system (Beckman Coulter, Brea, CA, USA). Finally, a hematological analysis was performed in the 28th week of the experiment.

Statistical analysis

Statistical analysis was conducted to evaluate the differences between DMBA-induced and xenograft-induced tumor weights, as well as changes in red and white blood cell counts over the experimental timeline. Tumor weights were analyzed using the Mann-Whitney U test due to the non-parametric distribution of the data. Hematological data were assessed using one-way ANOVA with repeated measures, followed by Tukey’s post hoc test for pairwise comparisons. Results were considered statistically significant at a p-value <0.05. All statistical analyses were performed using GraphPad Prism version 9.0 (GraphPad Software, San Diego, CA, USA).

## Results

How the animals behaved during tumorigenesis

Initially, 36 four-week-old female Wistar rats were included in the study. At eight weeks of age, the animals were ovariectomized to induce iatrogenic estrogenopenia. All rats survived the ovariectomy surgeries. At 10 weeks of age, simultaneous tumorigenesis was performed with DMBA on the right side of the chest wall and the MCF-7 tumor cell line on the left. The animals tolerated both surgeries and all infusions well. During the first 10 days after the injections, six animals developed ulcers at the DMBA injection site. Initially, these animals were placed alone in cages to monitor their health. A veterinarian evaluated them, and it was considered appropriate to exclude them from the experiment for two reasons: to prevent possible infectious spread to other healthy ones and because the additional inflammation clearly affected their immune status, which could affect the growth of the tumors.

All other animals remained healthy, active, and alert throughout the experiment. Observations of food and water consumption were consistent over the five-month study period. The baseline weight of the animals at the start of the study was 175.83±10.63 g, which increased to 235.43±18.82 g by the end of the study, reflecting a weight gain of 24.87±7.19 g across all groups over the 90 days following tumor induction. Weekly evaluations were conducted to monitor the development of tumor masses.

Regarding palpation, throughout the experiment, no tumors were detected at the DMBA injection sites. On the contrary, at the injection points of the cell line, tumors larger than 1 mm³ were detected five weeks after the injection and remained palpable throughout the experiment. After the completion of the experimental procedure, an autopsy was performed on the animals. Postmortem, tumors were found at the DMBA injection sites that were not externally palpable and were smaller than 1 mm³. All tumors were removed and weighed accordingly. DMBA tumors were found to be smaller than cell line tumors.

More analytically, DMBA-induced tumors weighed 22.96±3.97 g. Cell line-induced tumors weighed 55.06±2.92 g. The difference in weight was found to be non-parametric but statistically significant, with a p-value <0.05. Comparing the differences in tumor weights, the z-score is -6.6456 and the p-value, which is calculated <0.00001 (smaller than the significance level of 0.05), indicates a significant difference between the two groups being compared. Table 1 presents measurements for the initial and final body weight of the rats, the body weight increase during the course of the experiment, and the tumor weights of both the DMBA- and cell line-induced tumors.

**Table 1:** Animal weight gain and tumor observations during the course of the experiment. The data are represented as mean±SD (n=30). DMBA: 7,12-dimethylbenz(a)anthracene; CL: cell line.

Variable	Experiment rats
Initial body weight (g)	175.83±10.63
Final body weight (g)	235.43±18.82
Body weight increase (%)	24.87±7.19
Tumor weight DMBA (g)	22.96±3.97
Tumor weight CL (g)	55.06±2.92

Red blood cell (RBC) count was taken and measured in three time periods. T1 was on the 12th week of the experiment. The count was 7.95±0.75x10^6^/mm³ (CI: 0.13). T2 was on the 20th week of the experiment, and the RBC count was 7.78±0.76x10^6^/mm³ (CI: 0.13). Finally, T3 was on the 28th week of the experiment, shortly before the rats were euthanized and an autopsy was performed for findings related to the experiment. The RBC count was 7.81±0.80x10^6^/mm³ (CI: 0.14). As can be seen by the measurements that are summarized in Table 2, the RBC count remained stable in all three time periods.

**Table 2:** RBC and WBC count. RBC and WBC measurements during the course of the experiment. T1 was at 12 weeks, T2 was at 20 weeks, and T3 was at 28 weeks. The data are represented as mean±SD (n=30). CI refers to the 95% confidence interval of the mean. Reference value for RBC count: 7.0–9.0×10⁶ cells/mm³ and WBC count: 4.0–12.0×10³ cells/mm³. RBC: red blood cell; WBC: white blood cell.

Variable	T_1_	T_2_	T_3_
RBC count	7.95±0.75x10^6^/mm³ (CI: 0.13)	7.78±0.76x10^6^/mm³ (CI: 0.13)	7.81±0.80x10^6^/mm³ (CI: 0.14)
WBC count	3.94±0.56x10^6^/mm³ (CI: 0.10)	9.97±1.62x10^6^/mm³ (CI: 0.29)	2.19±0.41x10^6^/mm³ (CI: 0.07)

Lastly, white blood cells (WBCs) were also taken and measured in the same three time periods as the RBCs. At time period T1, the count was 3.94±0.56x10^6^/mm³ (CI: 0.10). At time period T2, the WBC count was 9.97±1.62x10^6^/mm³ (CI: 0.29). Finally, at time period T3, the WBC count was 2.19±0.41x10^6^/mm³ (CI: 0.07). From the measurements that can be seen in Table 2, there is an increase in WBC counts initially due to immunoreaction but as the experiment progresses and time passes there is also a noticeable drop, due to leukopenia.

## Discussion

When conducting tumor-induced experiments, in the context of a wider study for tumor formation and treatment, the success of the experiment depends entirely on the induction of tumorigenesis. The broader process of tumorigenesis is evaluated by the creation or not of tumors in the target organ, the time it takes to achieve carcinogenesis, the survival rate of the animals, and the strict compliance with what is submitted by scientific ethics and prescribed legislation for animal experiments [21-23].

One of the primary objectives of this study is to propose a model of tumorigenesis, resulting from a combination of two classical methods, to achieve carcinogenesis in as many animals involved in the experiment as possible, optimizing their living conditions throughout the experimental process. The methods used are a subcutaneous injection of DMBA into the mass glands and, simultaneously, an injection of MCF-7 secretory cell lines into the opposite row of the mass glands of the same animal.

It is highly debatable whether DMBA or the cell line achieved carcinogenesis in all animals. The majority of rats survived until the end of the experiment with a total duration of 10 months. An extremely important observation extracted from the experimental setup is that thymectomy was considered unnecessary to limit the perioperative morbidity of the animals. On one hand, it limits their quality of life, and on the other hand, it affects the sample size [24]. When it comes to secondary results, the ability of the animals to remain hemodynamically stable throughout the experiment was evaluated. Regarding their leukocyte status, initially, a mild leukocytosis was observed during tumorigenesis [25,26]. However, at the end of the experiment and after the tumors were palpable, the rats demonstrated leukopenia. This particular observation follows the findings of numerous studies in the international bibliography, according to which the use of BME is associated with immunosuppression [27].

While the study achieved its primary objectives, certain limitations should be acknowledged. First, the inability to palpate DMBA-induced tumors during the experimental period may hinder real-time monitoring of tumor development. This limitation could be addressed in future studies by altering the dosage or administration schedule of DMBA, as repeated or oral gavage methods may provide higher tumorigenesis rates according to existing literature. Furthermore, the limited sample size, with six animals excluded early due to health complications, may reduce the statistical power of the results. A larger cohort could improve the robustness and generalizability of the findings.

Another important limitation lies in the use of a single cell line (MCF7) for the xenograft model, which may not fully represent the heterogeneity of human breast cancer. Incorporating multiple cell lines with diverse oncogenic profiles would enable a broader investigation of tumorigenesis and therapeutic responses. Additionally, the study focused primarily on tumor induction without long-term observations of tumor progression or metastasis, which are critical for understanding the full spectrum of carcinogenesis and therapeutic applications. These factors should be addressed in subsequent research to enhance the translational relevance of the findings.

Finally, regarding tumorigenesis, which is also the research question, the data we received are controversial. Initially, 36 animals were used, of which 30 survived and all developed tumors. On the right side of the animals, i.e., at the DMBA injection site, during the 90 days of observation and weekly palpation, these animals did not appear to develop palpable tumors. From the existing literature, it appears that the one-time administration of an injectable carcinogen has lower rates of tumorigenesis than systematic daily oral gavage [28]. However, one-time administration was chosen in the context of reducing the interventions to avoid making the animals suffer while also simplifying the tumorigenic process. Nevertheless, at necropsies, and after the skin and subcutaneous layer were carefully removed, tumors were detected along the mass glands of the animals.

A reasonable question that arises is whether such a tumorigenesis model will be efficient in the context of an experiment with a pharmaceutical approach, given that in an experimental period of 90 days, it did not lead to palpable tumors. Could it be recommended as a safe option to working groups looking for the clinical detection of volume depletion to correlate it with drug treatment outcomes? Undoubtedly, it is an extremely interesting question, and further targeted research is needed to answer it and to find its practical application in future animal experimental setups. On the other hand, cell line tumorigenesis has led to rapidly detectable and clinically palpable tumors.

## Conclusions

The purpose of this study is to propose a mechanism of simultaneous tumorigenesis with a cell line and a local carcinogen to optimize the oncogenetic mechanisms in breast study experiments. The results showed 16.7% animal mortality in the initial stages of experimentation and then 100% induction of palpable tumors. The design of the said experiment cannot answer whether the simultaneous application of two methods worked synergistically; however, without a doubt, the positive results that were obtained leave room for additional research on this approach in the future.
